# Cryptococcal Meningitis in an Immunocompetent Patient With an Initially False-Negative Cerebrospinal Fluid Analysis: A Case Report

**DOI:** 10.7759/cureus.103252

**Published:** 2026-02-09

**Authors:** Sheau Wei Tan, Chuin Chi Yap, Sri Siva Shakti Ratanasamy

**Affiliations:** 1 Internal Medicine, Hospital Sultanah Nora Ismail, Batu Pahat, MYS

**Keywords:** cryptococcal meningitis, cryptococcoma, false-negative cerebrospinal fluid analysis, immunocompetent, leptomeningeal enhancement

## Abstract

Cryptococcal meningitis (CM) is traditionally associated with immunocompromised states, such as HIV/AIDS. However, its presentation in immunocompetent hosts is increasingly recognized and frequently poses a diagnostic dilemma. A 56-year-old immunocompetent male presented with obstructive hydrocephalus following a month of worsening headaches. Initial cerebrospinal fluid (CSF) analysis, including India ink, cryptococcal antigen, and cultures obtained during ventriculoperitoneal shunt placement, was unremarkable. Subsequent brain MRI demonstrated evolving leptomeningeal enhancement and small cerebellar nodules. Due to clinical overlap, the patient was empirically treated for tuberculosis but developed an adverse cutaneous reaction. Driven by worsening imaging findings, repeat CSF studies were performed; fungal culture and PCR eventually confirmed *Cryptococcus neoformans*. The patient was treated with induction amphotericin B and flucytosine, followed by maintenance fluconazole, resulting in a full clinical and radiological recovery. This case emphasizes that CM must remain a differential diagnosis in subacute meningoencephalitis or unexplained hydrocephalus, regardless of immune status. High clinical suspicion warrants serial CSF sampling and molecular diagnostics to prevent delayed treatment of this potentially fatal infection.

## Introduction

Cryptococcal infections remain a major disease burden globally, owing to their high mortality and morbidity rates, particularly among immunocompromised individuals such as those with HIV, solid organ transplants, or hematologic malignancies. *Cryptococcus neoformans*, which tops the WHO critical-priority group of the fungal priority pathogen list (FPPL) published in 2022, and *Cryptococcus gattii* are the two main species that are responsible for human infections [1]. Inhalation of these Cryptococcus species can result in a spectrum of disease, ranging from asymptomatic colonization to life-threatening pulmonary, neurological, and disseminated infections. It is estimated that every year, there are 152,000 cases of cryptococcal meningitis (CM) globally, with 112,000 cryptococcal-related deaths [2]. Although data regarding immunocompetent individuals is limited, recent literature suggests that cryptococcal meningitis in this population is not as rare as previously thought [3]. However, cryptococcal meningitis in immunocompetent individuals poses a diagnostic challenge because its clinical presentation can be ambiguous or gradual, and clinicians may not readily suspect it. This case report details a unique instance of CM in an immunocompetent patient, complicated by an initial false-negative cerebrospinal fluid (CSF) analysis. Typically, CM is diagnosed via brain imaging (CT or MRI) and a lumbar puncture with extended cultures. If fungal infection is suspected, a PCR may also be used. Clinicians should remain vigilant and repeat testing if symptoms persist despite negative results.

## Case presentation

**Figure 1:**
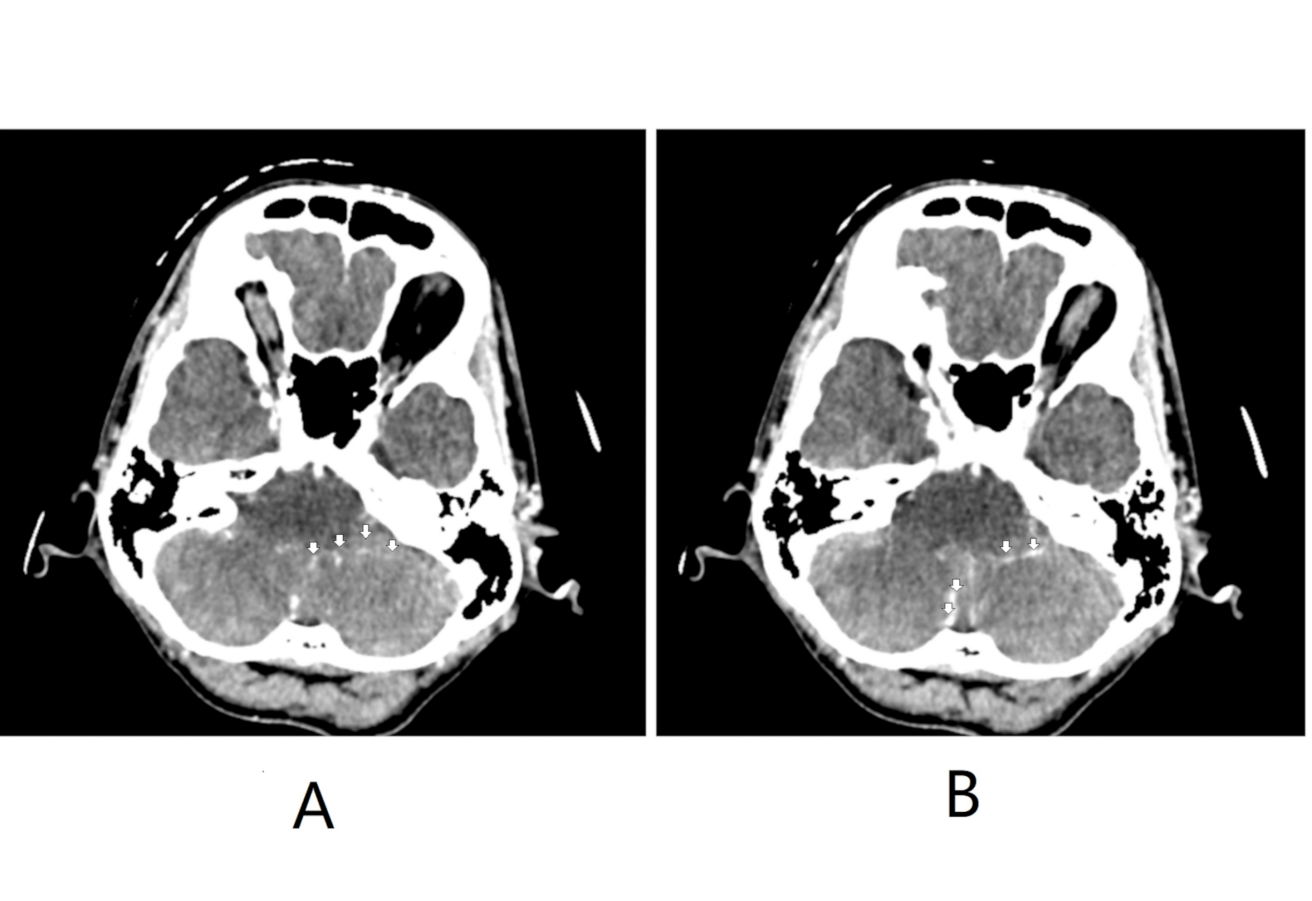
Axial contrasted CT of the brain taken at the level of the posterior fossa/cerebellum A: Multiple enhancing foci are seen within the cerebellar parenchyma (marked by arrows). The lesion adjacent to the aqueduct of Sylvius was 1.2x1.6 cm, causing compression and dilatation of the bilateral lateral and third ventricles. B: Leptomeningeal enhancement is seen at the midline anterior lobe of the lower cerebellum.

A 56-year-old school gardener with no known medical illness presented to the emergency department in September 2024 with a one-month history of progressively worsening generalized headache, associated with a three-day history of vomiting episodes. He also noted a loss of appetite and weight over the past two months. Otherwise, he had no blurring of vision, and a clinical examination found no neurological deficit. He had completed his childhood vaccinations and had no prior history of recurrent sinopulmonary or other infections. A contrast-enhanced CT of the brain noted obstructive hydrocephalus with an enhancing lesion at the midline anterior lobe of the cerebellum, adjacent to the aqueduct of Sylvius (Figure 1). A ventriculoperitoneal (VP) shunt was inserted the next day to relieve raised intracranial pressure. The CSF analysis sent intraoperatively came back normal (Table 1). The patient was then thoroughly investigated for malignancy. His tumor markers, including carcinoembryogenic antigen (CEA), cancer antigen 19-9 (CA 19-9), alpha-fetoprotein (AFP), and prostate-specific antigen (PSA), as well as CT scans of the thorax, abdomen, and pelvis, were all negative. He underwent an oesophagogastroduodenoscopy (OGDS), which showed reflux esophagitis with mild duodenitis; a colonoscopy only revealed hemorrhoids. His CSF cytology also found no evidence of malignant cells.

**Table 1:** Serial cerebrospinal fluid (CSF) analysis * Opening pressure was not documented during ventriculoperitoneal shunt insertion. † Opening pressure was not documented during ventriculoperitoneal shunt tap. § Tuberculosis GeneXpert® assay "-" represents tests not performed; MTB - *Mycobacterium tuberculosis*

CSF analysis	13/09/2024	17/10/2024	23/10/2024	15/12/2024
Opening pressure	Not documented*	Not documented†	15 cm H_2_O	13 cm H_2_0
Appearance	Clear and colorless	Clear and colorless	Clear and colorless	Clear and colorless
Cell count	0	0	Less than 5	1 (red blood cell)
Protein (g/L)	0.08	1.23	1.63	1.66
Glucose (mmol/L)	3.51	3.06	2.59	3.14
Globulin	Negative	Positive	Negative	Negative
India ink	Negative	-	-	-
Cryptococcal antigen	Negative	-	-	Negative
Culture	No growth	Yeast detected	-	No growth
Fungal PCR	-	Cryptococcal neoformans	-	-
Acid-fast bacilli	Negative	Negative	Negative	-
TB GeneXpert*®*§	-	-	Negative	-
MTB culture	-	No growth	No growth	-
Cytology	No malignant cells seen	Presence of some budding yeasts with no increase in accompanying inflammatory cells. No malignant cells seen.	No malignant cells seen	-

**Figure 2:**
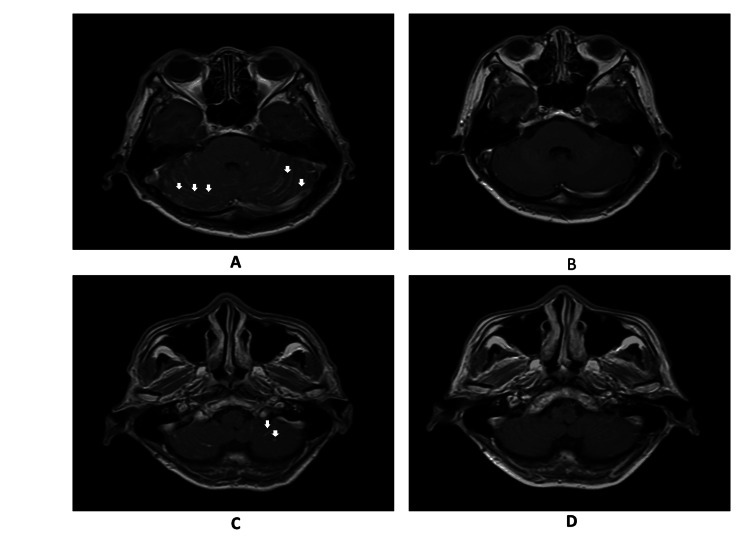
Axial brain MRI images before and after induction therapy A: Pre-induction axial T1-weighted image at the level of the posterior fossa demonstrating nodular leptomeningeal enhancement in bilateral cerebral hemispheres. B: Corresponding post-induction axial T1-weighted image showing markedly reduced leptomeningeal enhancement. C: Pre-induction axial T1-weighted image at a lower level demonstrating enhancing nodules at the base of the left cerebellar hemisphere. D: Post-induction axial T1-weighted image showing partial resolution following induction therapy.

In view of the unexplained obstructive hydrocephalus, an MRI of the brain was performed three weeks after the VP shunt insertion. The hydrocephalus had resolved post-VP shunt. However, there was increased leptomeningeal enhancement with multiple small nodular enhancements (<0.5 cm) in the bilateral cerebellar hemispheres (Figure 2). This raised a suspicion of tuberculous meningitis; therefore, a VP shunt tap was performed by the neurosurgical team. The second set of CSF analysis showed a raised protein level of 1.23 g/L and positive globulin (Table 1). At that point, the CSF culture result was pending due to yeast detection, and the sample was sent to a tertiary center for fungal PCR identification. However, this finding was not communicated to the clinical team managing the patient.

Following a multidisciplinary discussion among the neurosurgical, neuromedical, and radiological teams, a decision was made to empirically treat for tuberculous meningitis. The patient was started on rifampicin, isoniazid, pyrazinamide, and ethambutol. He was repeatedly admitted to the ward for adverse cutaneous reactions, and ethambutol was identified as the causative agent. During a review of previous investigations, his CSF fungal PCR was positive for *Cryptococcus neoformans*. MTB culture results were still pending. The diagnosis was revised to cryptococcal meningitis with cryptococcoma, and the patient was started on IV amphotericin B (0.7 mg/kg) with IV flucytosine. The antifungal treatment was complicated by renal impairment and transaminitis, which improved with dose adjustment and hydration. We did not rechallenge the anti-tuberculous agents due to the lack of supporting evidence for tuberculosis and a negative interferon-gamma release assay (IGRA). Both CSF MTB culture samples later returned with no growth. The patient completed six weeks of low-dose amphotericin B and two weeks of flucytosine; CSF clearance was achieved based on a lumbar puncture repeated at the third week of the induction phase. At the completion of the induction phase, an MRI of the brain showed markedly reduced bilateral leptomeningeal enhancement of the cerebellum, with minimal residual nodules at the base of the left cerebellum. The previously seen effacement of the fourth ventricle had also resolved. He was then started on the consolidation phase with oral fluconazole 400 mg daily for another eight weeks (unable to tolerate higher doses due to elevated liver enzymes). At the time of writing this article, he was on the maintenance phase with oral fluconazole 200 mg daily planned for 12 months and remained symptom-free.

Our patient had occupational exposure to pigeon droppings while working as a school gardener without wearing proper personal protective equipment. The European Confederation of Medical Mycology/International Society for Human and Animal Mycology/American Society for Microbiology (ECMM/ISHAM/ASM) guideline recommends evaluation for an undiagnosed immunodeficiency as part of the ten principles of management for cryptococcal meningitis [4]. Following a negative HIV test, he was screened for T-cell immunodeficiency. His CD4+ count was normal at 774 cells/µL, ruling out idiopathic T-cell lymphopenia. We then proceeded with a lymphocyte subset enumeration (TBNK) test during the induction treatment, which, interestingly, revealed a reduced total B-cell count of 80x10⁶/L (5%). This prompted further workup for humoral immunodeficiency. His IgA, IgG, and IgM levels were within normal limits at 4.04 g/L, 16.75 g/L, and 0.82 g/L, respectively (Table 2). An isohemagglutinin test revealed a low anti-B titre - IgM titre 1:16 and IgG titre 1:8 (Table 2). A detailed history found no significant history of recurrent sinopulmonary nor other types of infections, and no autoimmune, granulomatous, or lymphoproliferative diseases throughout his lifetime. During the eradication phase, his course was complicated by *Pseudomonas aeruginosa* bacteremia related to a central venous catheter, which was successfully treated. Subsequently, he had an uncomplicated varicella zoster infection during the consolidation phase as an outpatient. At that point, we were unable to confirm whether he truly had underlying primary immunodeficiency. Further follow-up with a repeated B cell count during the maintenance phase noted normalization to 236x10⁶/L, and a second set of blood immunoglobulin levels remained normal (Table 2). After a discussion with the immunologist, we concluded that this patient did not meet the criteria for common variable immunodeficiency [5]. Transient B cell lymphopenia in this case may be explained by acute infection and inflammatory responses, which can alter B cell production via several pathways, i.e., diverting lymphoid progenitors away from the B lineage, promoting apoptosis in developing B cells, and altering peripheral mobilization of developing B cells [6].

**Table 2:** Summary of investigations for immunodeficiency

Test	Date	Result	Unit	Reference range
HIV Ag/Ab	27/10/2024	Non-reactive		
CD4+	27/11/2024	774	Cells/µL	404 - 1612
CD8+		499	Cells/µL	220-1129
Isohemagglutinin titre (anti-B)				
IgM	23/12/2024	1:16		
IgG		1:08		
Lymphocyte subset enumeration (TBNK)				
T cell (CD3+)	3/12/2024	1252 (76%)	x 10^6^L	988-3912 (53-80%)
B cell (CD19+)		80 (5%)	x 10^6^L	130-716 (5-22%)
Natural killer (NK) cell		248 (15%)	x 10^6^L	227-1354 (8-37%)
T cell (CD3+)	15/1/2025	1555 (74%)	x 10^6^L	988-3912 (53-80%)
B cell (CD19+)		236 (11%)	x 10^6^L	130-716 (5-22%)
NK cell		275 (13%)	x 10^6^L	227-1354 (8-37%)
Immunoglobulin				
IgA	3/12/2024	4.04	g/L	0.63-4.84
IgG		16.75	g/L	5.4-18.22
IgM		0.82	g/L	0.22-2.40
IgA	8/1/2025	3.26	g/L	0.63-4.84
IgG		14.59	g/L	5.4-18.22
IgM		1.55	g/L	0.22-2.40

## Discussion

Diagnostic challenges in false-negative CSF and the role of repeated lumbar puncture

It is interesting to note that the first set of CSF analyses for our patient appeared normal despite the patient presenting with a complication of obstructive hydrocephalus. It is worth discussing the reasons that can explain its false negativity. India ink is a simple and quick test; however, sensitivity is only 86% even in experienced hands. If the colony-forming unit (CFU) level of Cryptococcus is <1000 per ml of CSF, this figure falls to 42% [7]. Cryptococcal antigen test (CrAg) is a more useful point-of-care diagnostic method. Since the CrAg lateral flow assay (LFA) was approved by the US FDA in 2011, it has been widely used and largely replaced the CrAg latex agglutination test [7]. It utilizes gold-conjugated monoclonal antibodies that can bind to cryptococcal capsular polysaccharide glucuronoxylomannan antigens, which are present in CSF or blood [8]. LFA comes in qualitative and semi-quantitative forms; our laboratory uses the former. LFA has a sensitivity up to 99.3% and specificity up to 99.1% in CSF samples in studies involving HIV-positive cryptococcal meningitis [9]. A more recent systematic review reported similar figures (99% for both sensitivity and specificity) for non-HIV CSF samples. [10]. A false-negative LFA in CSF can be explained by several reasons, such as early course of disease, low fungal burden, or cryptococcal species that are weakly encapsulated [11]. A paradoxical phenomenon called the "postzone effect" describes false-negative results that result from extremely high CrAg concentrations that prevent antigen-antibody crosslinking, which can be overcome by serial dilution [12]. It is interesting to note that even though LFA has lower sensitivity (96%) and specificity (96%) in serum, it can be detected weeks to months before meningitis symptoms develop. Serum CrAg titre also correlates with risk of cryptococcal meningitis and predicts mortality based on studies performed in HIV patients [7]. Therefore, in cases of high suspicion, it is worthwhile performing a plasma CrAg LFA.

CSF fungal culture remains the gold standard in the diagnosis of cryptococcal meningitis, but the long incubation period, from days up to two weeks, makes it difficult to expedite diagnosis and treatment initiation. Its sensitivity ranges between 82.4 to 94.2% [13]. In our case, the first set of CSF was only sent for routine culture with standard incubation of five days, which likely explains the negative result. A repeated CSF culture one month later detected yeast and was sent for species verification via a PCR test. A multiplex PCR assay for meningitis can diagnose a cryptococcal infection with a 96% confidence interval and 100% specificity [7]. Despite all the excellent tests available, a high index of clinical suspicion is crucial, and there is always a role for repeated lumbar puncture assessment if the diagnosis is unclear. 

Radiological features in cryptococcal meningitis, highlighting findings in immunocompetent hosts

Radiological findings of CM are highly variable and can be completely normal in certain cases. Commonly reported findings include hydrocephalus and meningeal enhancement [14], both of which were present in our case. However, these findings are also common in tuberculous meningitis.

Dilated Virchow-Robin (VR) spaces are frequently reported in CM regardless of the host's immune status, with predilection in areas like the basal ganglia, thalami, midbrain, periventricular, and cerebellar regions. These are characterized by a "soap-bubble appearance" made up of numerous small, round cysts filled with mucoid gelatinous material [14,15]. Foci of restricted diffusion, representing infarcts, are also commonly seen, usually in the basal ganglia [15]; however, it is important to consider other causes such as atherosclerosis and vasculitis. A "hazy brain base" is also described on MRI of CM patients as uniformly and symmetrically increased T2 signals, usually at the basal ganglia, midbrain, thalamus, and hypothalamus. This represents the sites where the perforating arteries are entering the brain [16]. The edema is caused by penetration of the fungal material along perivascular spaces into the basal brain [17]. 

Cryptococcomas, abscesses, enlarged choroid plexus, and ventriculitis are less commonly seen features [14]. It is interesting to note that cryptococcomas are more commonly seen in immunocompetent patients, such as in this case, as their formation requires an immunologic response from the host to encapsulate the cryptococci into these masses [16]. On MRI, these lesions appear hypointense on T1-weighted imaging (T1WI) and hyperintense on T2-weighted imaging (T2WI) and fluid-attenuated inversion recovery (FLAIR) sequences, with or without surrounding edema or rim enhancement [14,16].

## Conclusions

Cryptococcal meningitis in an immunocompetent host, while considered less frequent in occurrence than in the immunocompromised, poses a significant diagnostic challenge due to its gradual presentation, low index of clinical suspicion, and limitations of various diagnostic tests, as demonstrated in this case. The patient's initial false-negative CSF results necessitated repeated investigations. The ultimate diagnosis, facilitated by repeated lumbar puncture, detection of yeast on extended culture, and confirmatory fungal PCR, was critical in changing the management course.

Consequently, cryptococcal meningitis should be on the differential diagnosis for any patient presenting with subacute meningoencephalitis, unexplained hydrocephalus, or cerebral masses (cryptococcomas), regardless of their immune status. In the setting of high clinical and radiological suspicion, clinicians must maintain a proactive diagnostic approach, including the use of serial CSF sampling and advanced molecular testing when initial rapid tests are negative. Adopting this vigilant approach is vital to prevent significant morbidity and mortality from this treatable infection.
